# Supplementary figures and images for: Elucidating disease-associated mechanisms triggered by pollutants via the epigenetic landscape using large-scale ChIP-Seq data
Source: Epigenetics Chromatin. 2023 Sep 25;16:34. doi: 10.1186/s13072-023-00510-w (PMC10518938; doi:10.1186/s13072-023-00510-w)

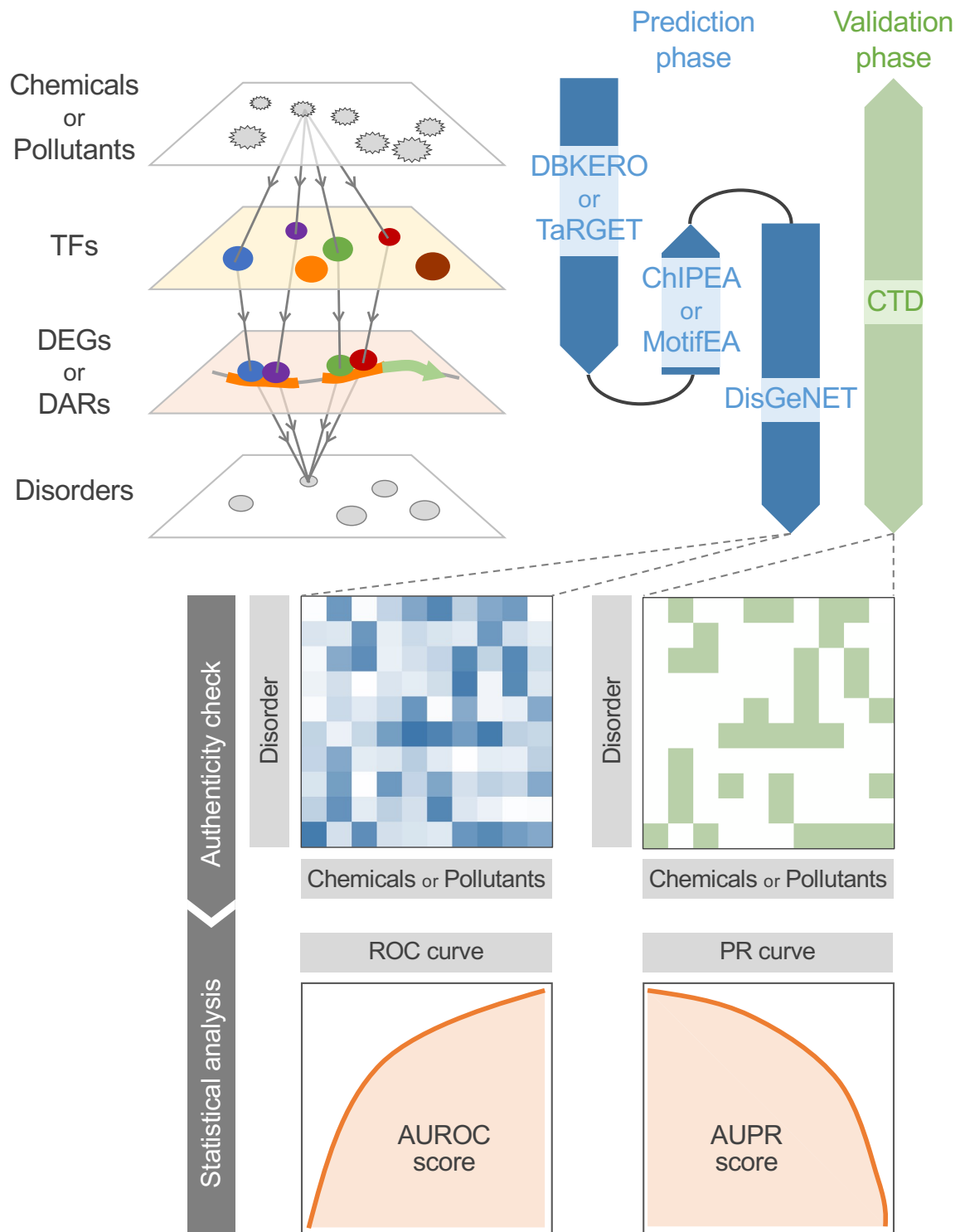

Supplementary Figure S1

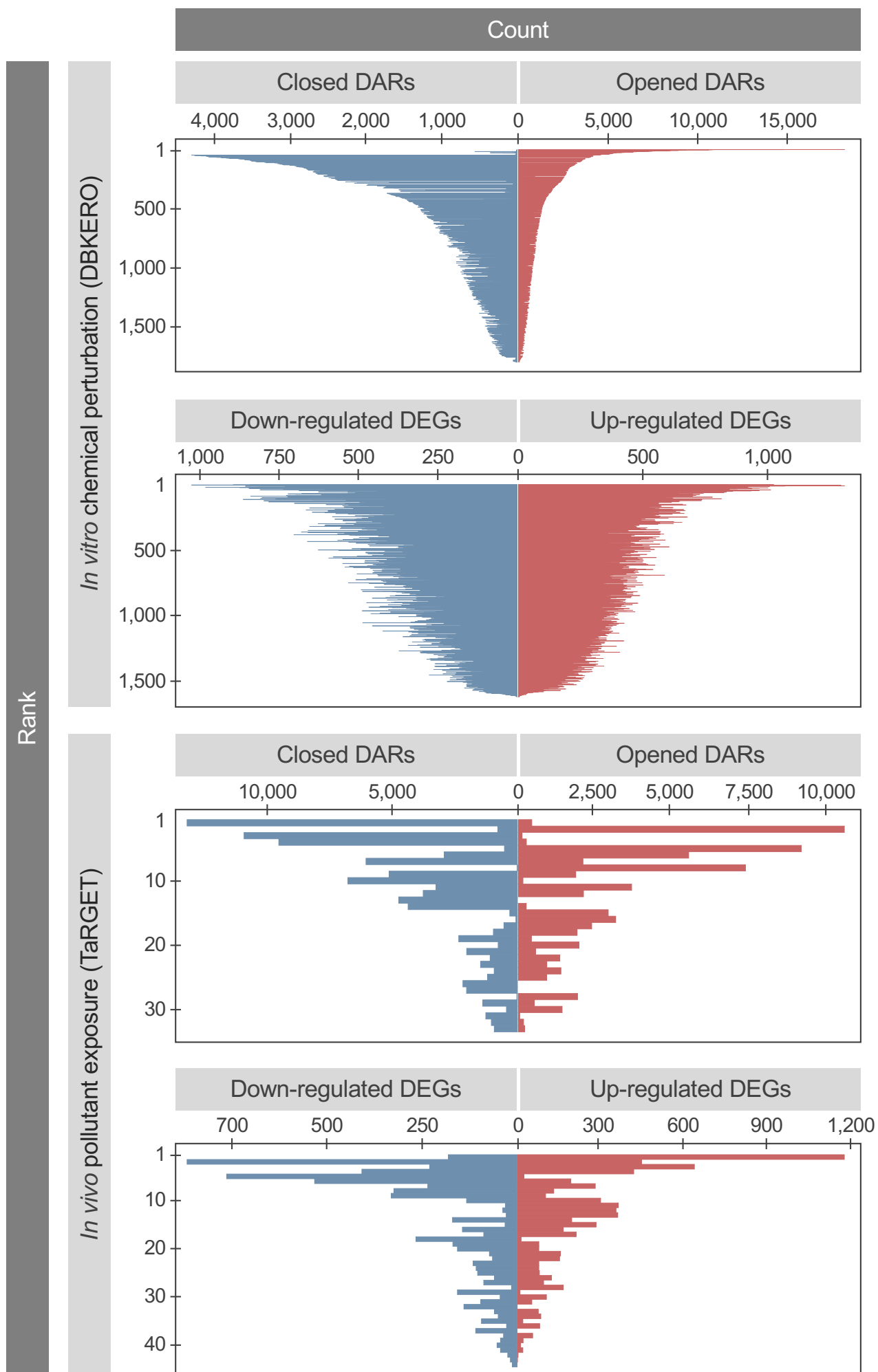

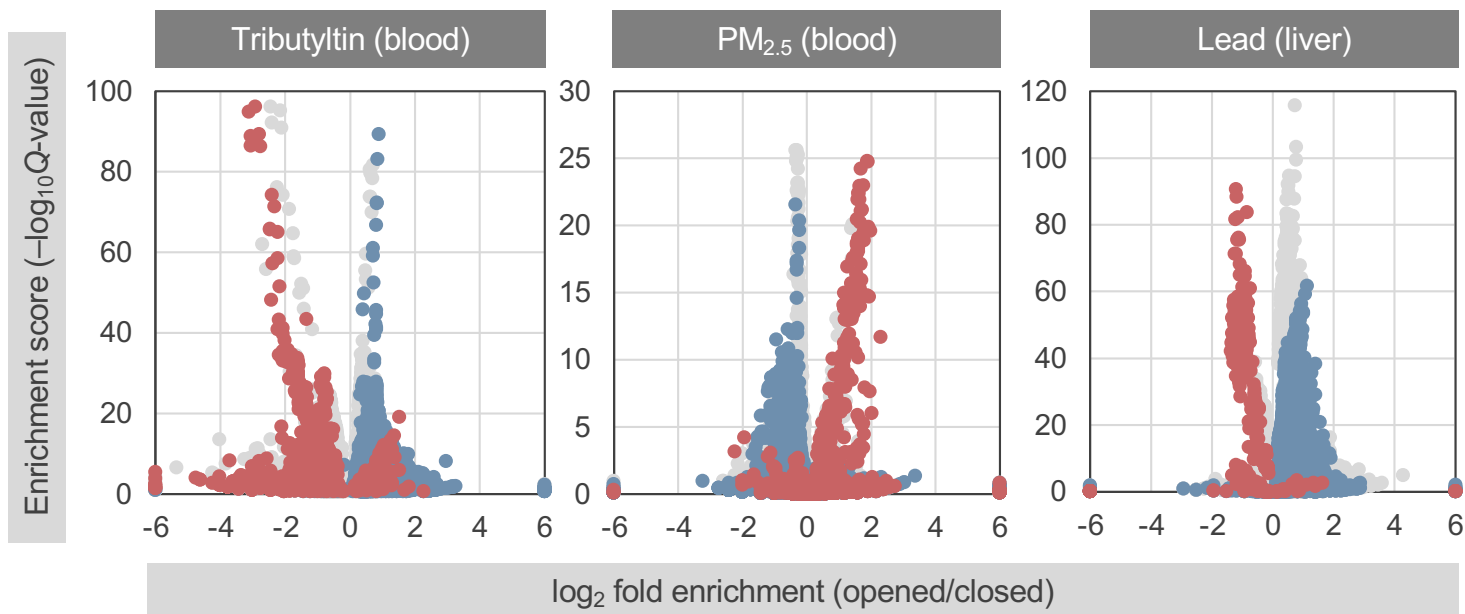

Supplementary Figure S3

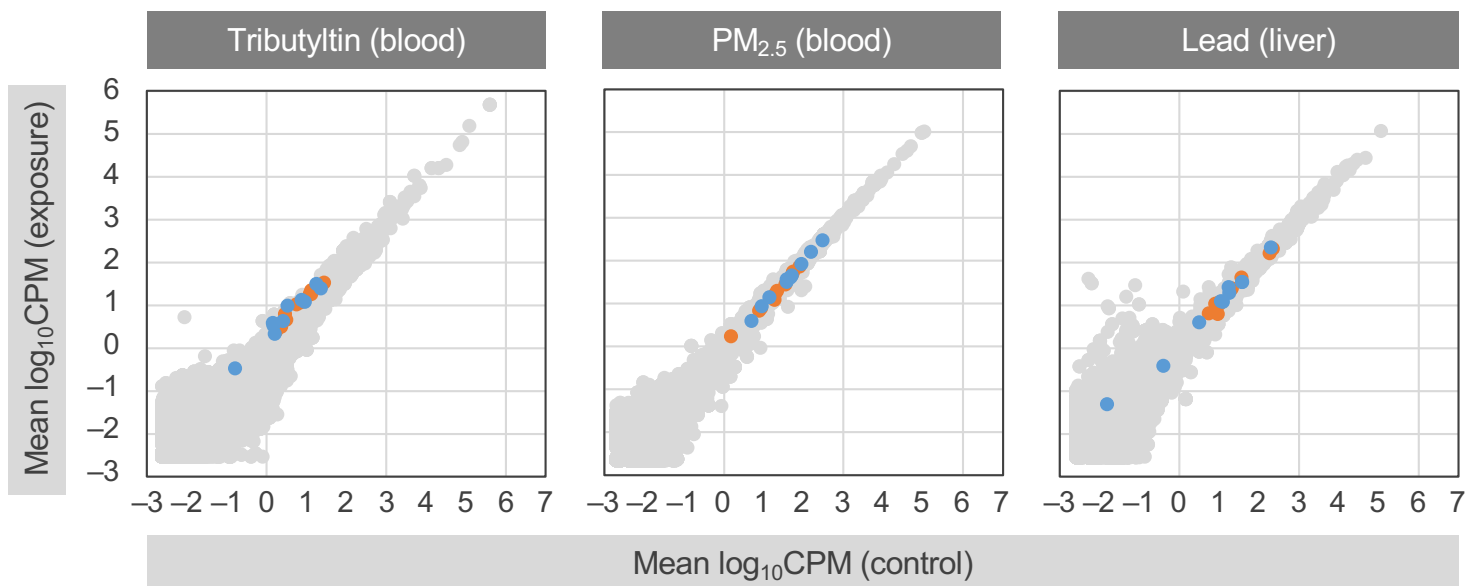

Supplementary Figure S4

Supplement: Supplementary file 1 — Additional file 1: Fig. S1. Workflow of the proposed approach. First, we detected the pollutant-induced DARs using ATAC-Seq data sets obtained from the TaRGET database (in vivo pollutant exposure). We then performed ChIPEA with pollutant-induced DARs to predict TFs that play a pivotal role in the pharmacological effects of pollutants on health. The prediction phase was completed upon construction of pollutant–TF–disorder triadic associations integrating TF-disease associations retrieved from DisGeNET with the pollutant–TF matrices obtained as outcomes of ChIPEA. Subsequently, in the validation phase, we checked the authenticity of the predicted pollutant–disorder associations (shown as blue-colored continuous matrix) by referencing known chemical–disorder associations from CTD (shown as green-colored Boolean matrix) and summarized the results as AUROC and AUPR scores. The in vitro chemical perturbation data set obtained from the DBKERO database was also used to evaluate the prediction accuracy of the proposed method. The performance of other methods that utilize chemically induced DEGs as input or employ motif-based enrichment analysis (MotifEA) rather than ChIPEA were simultaneously assessed using the same workflow. Fig. S2. Summary of the number of DEGs or DARs used as input to the enrichment analyses. Ranking plots are illustrated for individual chemical dosing or pollutant exposure conditions, and are sorted by the summation of the number of up- and down-regulated DEGs or opened and closed DARs. Fig. S3. Results of the Bisulfite-Seq enrichment analysis for three representative pollutants. Dots indicate individual Bisulfite-Seq peak sets from ChIP-Atlas. Peak sets obtained from Bisulfite-Seq on blood (in the case of TBT and PM2.5 exposure) and liver (in the case of lead exposure) samples are colored (hyper-methylated regions, red; hypo-methylated, blue). Positive and negative values of log2 fold enrichment (opened DARs/closed DARs; X-axis) indicate peak sets enri [file 13072_2023_510_MOESM1_ESM.pdf]
